# Supplementary figures and images for: Hemin Promotes Higher Effectiveness of Aminolevulinic-Photodynamic Therapy (ALA-PDT) in A549 Lung Cancer Cell Line by Interrupting ABCG2 Expression
Source: Med Sci (Basel). 2024 Nov 17;12(4):66. doi: 10.3390/medsci12040066 (PMC11587042; doi:10.3390/medsci12040066)

Figure S1. Western blotting of ABCB1 and ABCG2 proteins

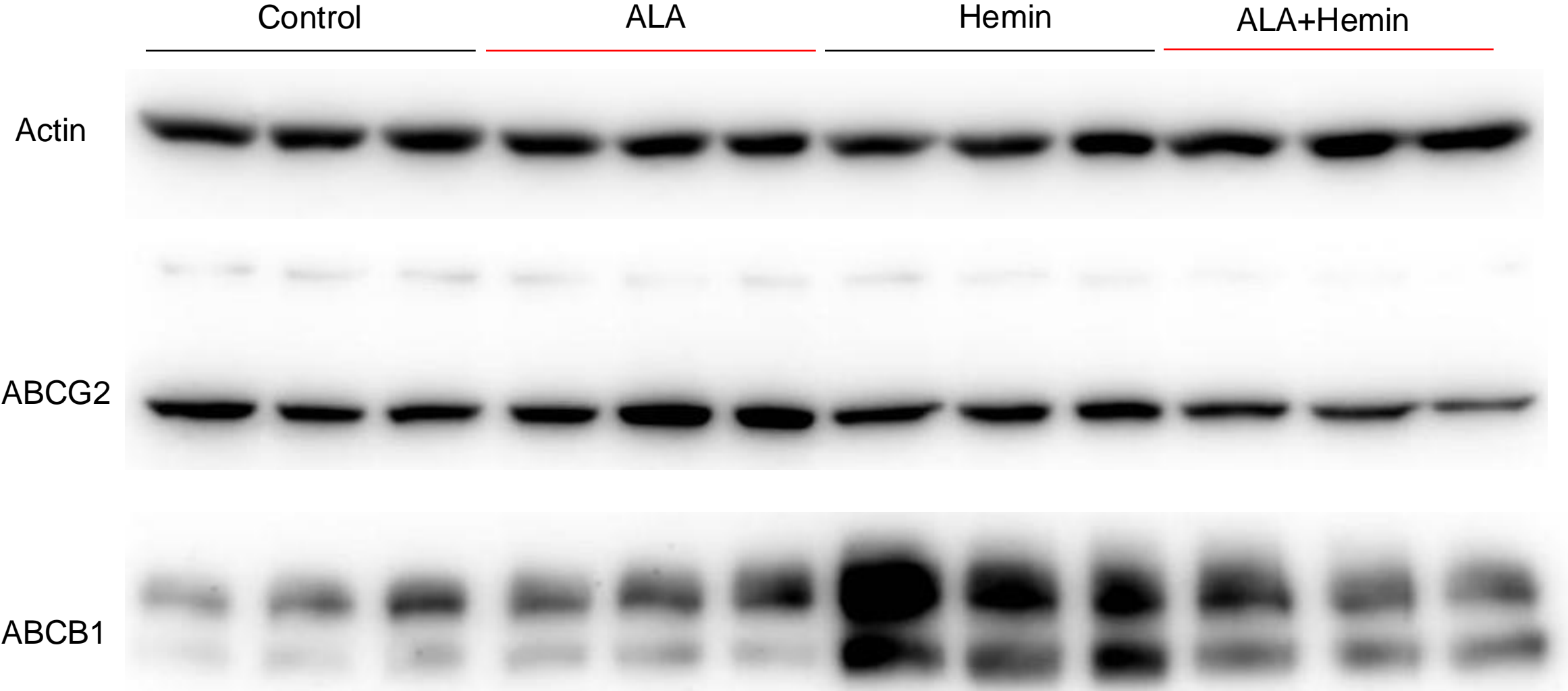

Supplement: Supplementary file 1 [file medsci-12-00066-s001.zip › medsci-3292212-supplementary.pdf]
